# Annotation Comparison Explorer (ACE): connecting brain cell types across studies of health and Alzheimer’s Disease

**DOI:** 10.1101/2025.02.11.637559

**Published:** 2025-02-12

**Authors:** Jeremy A. Miller, Kyle J. Travaglini, Tain Luquez, Rachel E. Hostetler, Aaron Oster, Scott Daniel, Bosiljka Tasic, Vilas Menon

**Affiliations:** 1. Allen Institute for Brain Science,; 2. Columbia University,

## Abstract

Single-cell multiomic technologies have allowed unprecedented access to gene profiles of individual cells across species and organ systems, including >1000 papers focused on brain cell types alone. The Allen Institute has created foundational atlases characterizing mammalian brain cell types in the adult mouse brain and the neocortex of aged humans with and without Alzheimer’s disease (AD). With so many public cell type classifications (or ‘taxonomies’) available and many groups choosing to define their own, linking cell types and associated knowledge between studies remains a major challenge. Here, we introduce Annotation Comparison Explorer (ACE), a web application for comparing cell type assignments and other cell-based annotations (e.g., donor demographics, anatomic locations, batch variables, and quality control metrics). ACE allows filtering of cells and includes an interactive set of tools for comparing two or more taxonomy annotations alongside collected knowledge (e.g., increased abundance in disease conditions, cell type aliases, or other information about a specific cell type). We present three primary use cases for ACE. First, we demonstrate how a user can assign cell type labels from the Seattle Alzheimer’s Disease Brain Cell Atlas (SEA-AD) taxonomy to cells from their own study and compare these cell type mappings to existing cell type assignments and cell metadata. Second, we extend this approach to ten published human AD studies which we previously reprocessed through a common data analysis pipeline. This allowed us to compare brain taxonomies across otherwise incomparable studies and identify congruent cell type abundance changes in AD, including a decrease in abundance of subsets of somatostatin interneurons. Finally, ACE includes translation tables between different mouse and human brain cell type taxonomies publicly accessible on Allen Brain Map, from initial studies in individual neocortical areas to more recent studies spanning the whole brain. ACE can be freely and publicly accessed as a web application (https://sea-ad.shinyapps.io/ACEapp/) and on GitHub (github.com/AllenInstitute/ACE).

## Introduction

Since their invention more than a decade ago, single cell (Tang *et al*., 2009) and single nucleus (Grindberg *et al*., 2013) RNA-sequencing technologies (sc/snRNA-seq) have revolutionized the definition of cell types from a largely qualitative to a highly quantitative concept. Large scale, community efforts from the Human Cell Atlas (Regev *et al*., 2017) and others now aim to comprehensively define and characterize all cell types in the human body. In the brain, cell types historically defined by their shape, firing properties, or expression of at most a few marker genes, can now be defined by the molecular content of their somas or nuclei. The Allen Institute has created foundational atlases characterizing brain cell types in the adult mammals (Yao *et al*., 2023; Yao, van Velthoven, *et al*., 2021; Bakken *et al*., 2021; Hodge *et al*., 2019; Tasic *et al*., 2018; Jorstad, Close, *et al*., 2023; Jorstad, Song, *et al*., 2023; Siletti *et al*., 2023), with more than 3000 cell types in human (Siletti *et al*., 2023) and more than 5000 cell types in mouse (Yao *et al*., 2023) defined to date, finding that molecularly-defined types from sc/snRNA-seq largely (but not entirely) align with historically-defined types and tend to be anatomically constrained. Cell type classifications (or ‘taxonomies’) have proven to be highly conserved across data collected on different platforms, across multiple data modalities, and even between species where few homologous marker genes show conserved patterns (Bakken *et al*., 2021; Hodge *et al*., 2019; Jorstad, Song, *et al*., 2023; Yao, Liu, *et al*., 2021). Sc/snRNA-seq can also identify changes in cell type abundance, cell state, or gene expression profiles in disease, including Alzheimer’s Disease (AD) (Gabitto *et al*., 2024; Mathys *et al*., 2023, 2019; Olah *et al*., 2020; Zhou *et al*., 2020; Lau *et al*., 2020; Leng *et al*., 2021; Morabito *et al*., 2021; Yang *et al*., 2022; Cain *et al*., 2023; Green *et al*., 2023), where several groups have identified novel microglial and other glial states associated with AD pathology (Morabito *et al*., 2021; Green *et al*., 2023; Deczkowska *et al*., 2018; Keren-Shaul *et al*., 2017). Collectively, these efforts present brain cells as a complex landscape of intersecting molecular cell types and cell states, which neuroscientists are slowly coming to understand.

With so many public cell type classifications available and many groups choosing to define their own, linking cell types and associated knowledge between studies remains a major challenge. Many studies define their own classifications using inconsistent nomenclature. For example, three recent studies that identified microglial types with increased abundance in AD defined them as Mic. 12/13 (Green *et al*., 2023), MG1 (Morabito *et al*., 2021), and Micro-PVM_3 - SEAAD (Gabitto *et al*., 2024) using different abbreviations for microglia and a different system for numbering cell types. Furthermore, cell typing papers often have different levels of resolution depending on the number of cells collected, the quality of the data, or the focus of the study. For example, these same microglial studies arrived at very different numbers of total microglial populations: 16, three, and six, respectively. Furthermore, many studies of disease break neurons into a handful of broad groups, while atlases in the healthy brain identify several dozen neuronal types per region. Public tools like MapMyCells (RRID:SCR_024672) and Azimuth (Hao *et al*., 2021) provide user-friendly interfaces for scientists to align data to reference taxonomies, and recent efforts have attempted to standardize nomenclature in brain cell types (Miller *et al*., 2020) for research groups committed to defining their own cell types. Even as these efforts gain traction, linking information from newer, larger studies to highly cited and well-annotated studies on focused brain regions and cell types remains a challenge.

To address these challenges, we have developed Annotation Comparison Explorer (ACE), a web and associated R shiny application for comparison of two or more annotations such as (i) cell type assignments (e.g., from different mapping/clustering algorithms), (ii) donor metadata (e.g., donor, sex, age), and (iii) cell metadata (e.g., anatomic location, QC metrics). ACE comes prepopulated with several highly requested translations between published cell type taxonomies in mouse and human brain, and between different studies of AD in human dorsolateral prefrontal cortex (DLPFC), and also allows users to provide their own tables for comparing annotations. Here we describe ACE functionality and present common use cases. ACE is freely available for use and collaboration as a web application (https://sea-ad.shinyapps.io/ACEapp/) and on GitHub (github.com/AllenInstitute/ACE).

## Results

### How does ACE work?

ACE compares cell type annotations across datasets in three sequential steps. First, a data set must be selected, either by choosing a pre-defined comparison of public mouse/human cell type taxonomies or by uploading user data. Second, the data set can optionally be filtered to focus on specific annotations. Finally, filtered data can be explored through a variety of interactive visualizations and statistics. ACE also contains an informational pane with links to additional resources for help or to contribute, including an informational webinar and an extensive user guide detailing each component of ACE and listing additional use cases. In the example in Supplemental Figure 1, eight pyramidal neurons are initially classified into two groups based on size. Addition of a ninth neuron leads to a reclassification into three groups, resulting in two different size annotations for the original eight neurons. After saving in a simple format, ACE can show how size classifications change between the two annotations using river plots, revealing shifts in the definitions of size categories.

**Figure 1:**
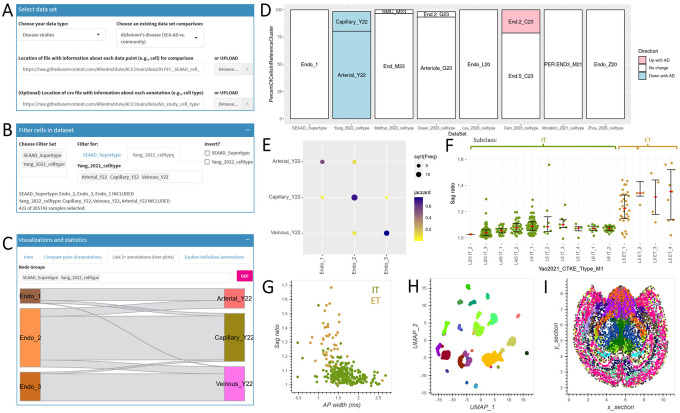
Overview of ACE functionality. **A-C)** Screenshots of the three main components of ACE as applied to comparison of endothelial cell types between two studies of AD. The top panel (**A**) allows a user to select from a predefined data set (as shown here) or upload their own. The middle panel (**B**) allows a user to filter data points within the selected data set based on one or more annotation columns, in this case by only include cells labeled as endothelial types in the SEAAD and Yang_2022 studies. The bottom panel (**C**) includes tabs for all visualizations and analysis performed by ACE on the filtered dataset, in this case showing the relationship between annotations of three endothelial cell types in SEAAD and Yang_2022. **D-I)** Visualizations available in ACE. **D**) Custom bar plots showing the relationship between single value (Endo_1) in one annotation (SEAAD_Supertype) and values in one or more other annotations (cell type assignments from other studies), and color-coding by whether these values show a change in abundance with AD. **E**) Confusion matrices showing the Jaccard similarity between all values in a pair of annotations, in this case showing the same information as in **C**. **F**) Bee swarm plots showing any numeric property (Sag ratio) divided by values of one annotation (Yao2021_CTKE_Type_M1) and color-coded by another (Subclass). **G-I**) Scatterplots showing the relationship between two numeric annotations such as electrophysiological parameters (**G**), UMAP coordinates (**H**), or physical cell locations (**I**), and color-coding by a categorical annotations (Subclass).

The “Select dataset” panel in ACE allows users to choose pre-built annotation comparison tables or upload their own (Figure 1A). Pre-built tables include comparisons for disease studies (AD), human and mouse cell type classifications, and patch-seq data (linking electrophysiological properties to gene-based cell types). All pre-built tables are available on GitHub and include an informative project description (see also Supplemental Table 1). For user-provided data, ACE accepts two input matrices: a required data (e.g., cell) table and an optional annotation table. The data table encodes data points (e.g., cells) as rows and various cell annotations (e.g., cell type assignments, anatomic structures, QC metrics) as columns and is typically shared as a csv (or gzipped csv) file. The annotation table provides additional information about each annotation like more detailed cell type descriptions and information about abundance changes with disease (encoded in the “direction” column). If provided, the order of annotations in this file dictates the ordering annotation is shown in ACE visualizations.

The “Filter cells in dataset” panel allows users to restrict the input data to specific annotations (e.g., endothelial cells rather than all cells) (Figure 1B). The “Choose Filter Set” box lets users select one or more metadata columns for filtering, with autocomplete functionality to aid in selection. For each selected variable, the “Filter for” box allows filtering by specifying included values (for categorical data, with an option to invert the selection) or setting a numeric range. Details about the current selection are also shown in the app.

The “Visualization and statistics” panel provides a variety of interactive options for exploration of the relationships between selected data points. For example, river plots show relationships between two or more categories arranged in columns. The “rivers” connecting adjacent columns show the number of items (e.g., cells) shared between categories, with thicker rivers representing larger overlaps. After filtering for endothelial cells (Figure 1B), a clear relationship between SEA-AD supertypes (Gabitto *et al*., 2024) and reported arterial, capillary, and venous vascular cell types (Yang *et al*., 2022) can be seen (Figure 1C), suggesting obvious improvements to future cell type annotations. ACE also provides novel functionality for focused exploration of individual annotations; for example, arterial cells represent a distinct cell population across multiple studies (Figure 1D). A confusion matrix can be used to show the correspondence between two different sets of classifications (in Figure 1E, cell type assignments from two different studies). The matrix displays how many items (cells) assigned to a particular category in one classification are assigned to each category in the other classification, and the size and color of points can be used to encode different types of information like Jaccard similarity, fraction representation, or cell counts. When comparing two annotations, confusion matrices and river plots show the same information in different ways.

ACE can also visualize numeric variables (Figure 1F–I). For example, electrophysiological differences between intratelencephalic (IT) and extratelencephalic (ET) cells is well established with ET neurons having a higher Sag ratio than IT neurons in humans (Kalmbach *et al*., 2021). By exploring an available table of patch-seq data collected in mouse primary motor cortex (Scala *et al*., 2021) and restricting the view to these two subclasses (ET and IT), this difference is clearly consistent between all ET and IT cell types (Figure 1F), and matches previous reports in humans (Kalmbach *et al*., 2021). Additional separation between mouse ET and IT cells can be seen when comparing Sag ratio (higher in ET) and action potential (AP) width (higher in IT) using a scatter plot (Figure 1G). While not its primary use, ACE’s “Compare numeric annotations” can also be used to overlay annotations on latent representations of single cell RNA-sequencing data (Figure 1H) or on physical cell locations in tissue slices (Figure 1I). For example, mouse cell types show clear spatial patterning in the brain, as previously reported (Yao *et al*., 2023; Zhang *et al*., 2023).

### Use Case 1: Comparing user-provided clustering and MapMyCells mapping results

SIngle cell and single nucleus RNA-seq has become the standard method for cell type identification and characterization not only in the brain, but across all organ systems (Regev *et al*., 2017), both in health and in disease. While most studies still define their own cell type classifications, large-scale efforts to define integrated reference atlases in brain and other organ systems (e.g., lung (Sikkema *et al*., 2023)), provide comprehensive transcriptomic cell type definitions in the context of extensive multi-modal knowledge. In human and mouse neocortex, for example, morphoelectric properties of cells can be inferred by aligning Patch-seq studies (Kalmbach *et al*., 2021; Scala *et al*., 2021; Gouwens *et al*., 2020; Berg *et al*., 2021; Chartrand *et al*., 2023; Lee *et al*., 2023) with existing cell type taxonomies, and spatial locations of all mouse brain cell types have been established using spatial transcriptomics (Yao *et al*., 2023). The Allen Institute has developed several resources for cross-study analysis and label transfer to facilitate the transfer of this knowledge to other groups, including: (1) multiple high-quality and well-annotated cell type taxonomies (Tasic *et al*., 2018; Hodge *et al*., 2019; Yao, Liu, *et al*., 2021; Yao, van Velthoven, *et al*., 2021; Yao *et al*., 2023; Bakken *et al*., 2021; Jorstad, Song, *et al*., 2023; Jorstad, Close, *et al*., 2023; Gabitto *et al*., 2024; Siletti *et al*., 2023) stored in standard formats and with public data visualization tools (e.g., the Allen Brain Cell Atlas (RRID:SCR_024440)); (2) interactive and code-based algorithms for assigning cell types from these taxonomies to user data (e.g., MapMyCells), and (3) tools for visualization and exploration of cell type assignments across studies such as ACE.

**Figure 2:**
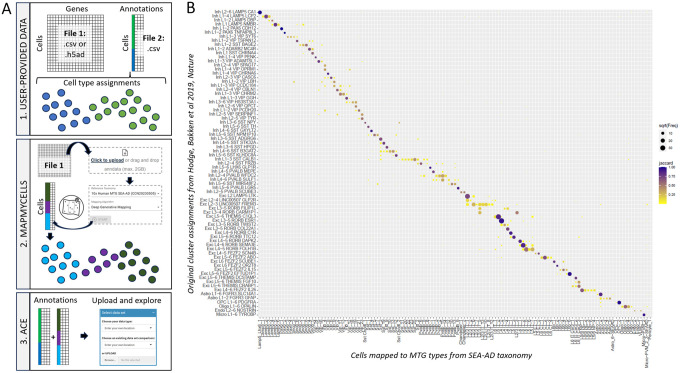
Comparison of user and reference brain cell types using MapMyCells and ACE. **A**) Overview of user workflow for a code-free comparison of user-provided gene expression data and associated cell annotations with cell type mapping results using MapMyCells. **B**) Confusion matrix after using this workflow to map gene expression data from Hodge, Bakken et al 2019 to the SEA-AD taxonomy. These results are provided as a predefined table in ACE (“Middle temporal gyrus (initial studies)”), with details for mapping Hodge to SEA-AD available on Allen Brain Map at https://portal.brain-map.org/atlases-and-data/bkp/mapmycells/mapmycells-use-case-single-nucleus-rnaseq-from-human-mtg.

Here we demonstrate how to identify the Allen Institute-hosted brain cell types associated with user-provided single cell/nucleus RNA-seq data and compare these results with user metadata without the need to code (Figure 2A). First, collect a cell by gene matrix in a standard format (h5ad or csv) and a cell annotation table that includes pre-defined cluster calls. Next, run MapMyCells to transfer cell types from your taxonomy of interest using one of three available algorithms. Detailed instructions for running MapMyCells are available on Allen Brain Map, but this involves uploading a cell by gene matrix, clicking a few options in a graphic user interface (GUI), and then downloading and unzipping results. Third, join the mapping results downloaded from MapMyCells with the initial cell annotation table to create a single file containing all cell annotations. Finally, upload this file (along with any relevant cell type annotations) into ACE and explore.

To demonstrate the utility of this workflow, we took data from an early classification of MTG that has been used for patch-seq mapping (Hodge, Bakken, et al 2019; Ref (Hodge *et al*., 2019)) and transferred labels from the more recent SEA-AD study, which includes approximately 100-fold more cells. Reassuringly, we find good correspondence between original cluster assignments and mapped cell types, with the great majority of cells from the same cluster mapping to one or very few SEA-AD types (Figure 2B). Areas of the most disagreement match expectations, with additional resolution in several undersampled inhibitory (e.g., SST) and deep-layer excitatory (e.g., L5/6 NP) types, particular confusion in gradient cell types (e.g., L2/3 IT cells, Pvalb interneurons), and lack of representation for most types specifically found in age and AD (cell types with -SEA-AD suffix). As with the studies discussed in the next use case, these studies used different experimental methods, and together validate the utility of mapping of this workflow of applying MapMyCells followed by ACE for cell type annotation and visualization.

### Use Case 2: Comparing brain cell types in AD across 11 studies

Multiple published snRNA-seq studies describe cellular and molecular changes in AD (Mathys *et al*., 2023, 2019; Olah *et al*., 2020; Zhou *et al*., 2020; Lau *et al*., 2020; Leng *et al*., 2021; Morabito *et al*., 2021; Yang *et al*., 2022; Cain *et al*., 2023; Green *et al*., 2023), but they all use different names for neuronal and glial types, making cross-study comparisons challenging. In a previous effort (Gabitto *et al*., 2024), we harmonized snRNA-seq data and associated donor metadata across 11 such studies of AD including SEA-AD and 10 community-based data sets. This involved reprocessing raw sequencing data though the SEA-AD analysis pipeline and then mapping cells to SEA-AD cell types.

Here we extend this work by linking mapping results with initial cell type assignments and reported changes in abundance with AD. Original cell type classifications were collected and linked to the mapped cell types using unique cell identifiers, retaining only cells with both SEA-AD and initial assignments. To link community annotations with individual cells from SEA-AD, we determined the proportion of cells from each SEA-AD supertype originating from each assigned cell type in that study and then probabilistically assigned cell types to each cell based on these proportions. Cells with SEA-AD supertypes not found in a given study were classified as “noMappedCells,” and all of these annotations are saved in the cell annotation table. Finally, we reviewed each study to determine the direction of abundance change with AD and recorded this information, along with additional cell type metadata, in an annotation information table available in the web version of ACE.

**Figure 3:**
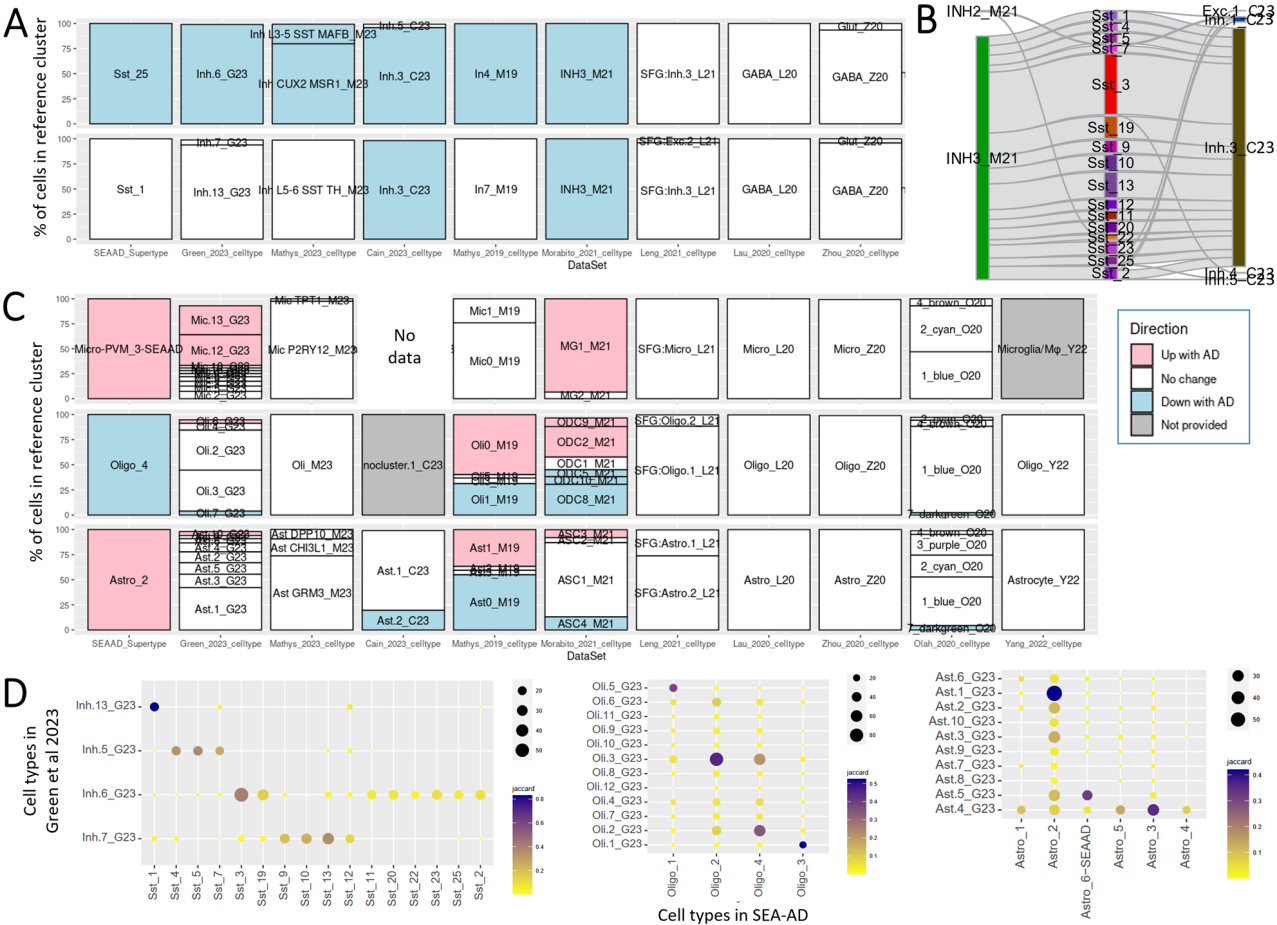
Comparison of cell types across multiple studies of AD. **A)** Cell type assignments in eight other studies of AD for neuronal cells assigned to Sst_25 (top) and Sst_1 (bottom) in SEAAD. Green_2023, Mathys_2023, Mathys_2019, and SEAAD show a consistent decrease in abundance in AD for cells in Sst_25, but not Sst_1 (legend in **C**). **B**) River plots comparing Sst cells in SEAAD (middle column), with Morabito_2021 (left) and Cain_2023 (right) shows that both studies have substantially lower resolution, with most Sst cells mapping to a single type. **C**) Cell type assignments in ten other studies of AD for glial cells assigned to Micro-PVM_3-SEA-AD (top), Oligo_4 (middle), and Astro_2 (bottom) in SEAAD. Abundance changes for microglia are consistent across studies, but less robust than changes in Sst_25, while abundance changes in other glial types vary across studies. **D**) Confusion matrices showing cell type alignment between SEA-AD and Green et al 2023 for Sst neurons (left), oligodendrocytes (middle), and astrocytes (right). SEA-AD identifies more neuronal types and fewer glial types than Green et al 2023 and there tend to be less confusion in neuronal cell type assignments.

One key finding from our previous work is that SEA-AD and the two largest studies of dorsolateral prefrontal cortex (DFC) (Mathys *et al*., 2023; Green *et al*., 2023) show common abundance changes in AD, including loss of supragranular somatostatin (SST) neurons and gain of disease-associated microglia (Gabitto *et al*., 2024). We used ACE to explore abundance changes with AD across the remaining studies of AD, which were underpowered for direct statistical assessment using an integration approach. These same SST neurons showed consistent cell loss in donors with AD pathology in several other published studies; for example, SST_25 abundances decreased with AD six of the nine studies that included neurons (Figure 3A, top row). Interestingly, other SST neurons that we identified as relatively spared consistently failed to show decreases in AD with disease across published studies (e.g., SST_1, Figure 3A, bottom row). The only two studies showing decreases in SST_1 abundance in AD are studies that group nearly all SST cells into a single type, suggesting study differences in this case are due to lack of cell type resolution (Figure 3B). Changes in abundance of other cell types show less reproducibility between studies. Disease associated microglia (Micro-PVM_3-SEAAD, Figure 3C, top row) show increased abundance in two other studies, but are not reported as increased in several others, potentially due to lower statistical power (Gabitto *et al*., 2024). Changes in other glial types are less consistent, with oligodendrocyte and astrocyte types showing decreased and increased abundance with AD in SEA-AD corresponding to types showing a variety of patterns elsewhere (Figure 3C, bottom rows). These inconsistencies in glial results may be due to differences in how cell types are defined; SEA-AD defined more neuronal types, whereas other studies (e.g. (Green *et al*., 2023)) defined more glial types, and cell type definitions tend to be better aligned for neuronal than non-neuronal types across studies (Figure 3D). These differences could also reflect discrete and more continuous identities among neuronal and non-neuronal cells, respectively. With more continuous identities in non-neuronal cells, there are a greater number plausible boundaries that are reasonable, which is influenced by decisions in dataset pre-processing, on which genes are considered through feature selection, on whether and how donor and library effects are modeled, by what clustering parameters are used, and on whether orthogonal experimental data were considered.

Together these results highlight the importance both of aligning on a consistent cell type nomenclature when defining cell types and of having tools for visualizing cross-study patterns when studying abundance changes in AD.

### Use Case 3: Translating between reference brain cell type taxonomies

The Allen Institute has developed multiple high-quality and well-annotated cell type taxonomies (Tasic *et al*., 2018; Hodge *et al*., 2019; Yao, Liu, *et al*., 2021; Yao, van Velthoven, *et al*., 2021; Yao *et al*., 2023; Bakken *et al*., 2021; Jorstad, Song, *et al*., 2023; Jorstad, Close, *et al*., 2023; Gabitto *et al*., 2024; Siletti *et al*., 2023) over the past decade, starting with focused studies in individual brain areas and gradually increasing in scope as technologies have improved over the past decade. Most of these studies are accompanied by web applications on Allen Brain Map that are tied to specific cell type taxonomies. A major challenge is linking cell type names and associated knowledge from earlier studies to more recent studies spanning the whole brain both for scientists within the Allen Institute and for the neuroscience community at large. For example, we receive many questions asking how to link cell types from our initial, widely used neocortical studies (Hodge *et al*., 2019; Tasic *et al*., 2018) to more recent studies of the same brain regions that include more data (human) or span the whole brain (mouse and human). A complete list of transcriptomics brain atlases created by the Allen Institute with associated data exploration tools is shown in Table 1.

To facilitate translation of cell type names and (to some extent) knowledge between studies, ACE includes several annotation tables translating cell types between brain atlases, which can be accessed by choosing “Mouse Cell Type Classification” or “Human Cell Type Classification” as the data type in ACE. This section describes several questions involving translation between reference cell type taxonomies that can be addressed using ACE.

#### Which cell types in the whole mouse brain correspond to a specific cell type in mouse whole cortex & hippocampus, and what do we know about them?

**Figure 4:**
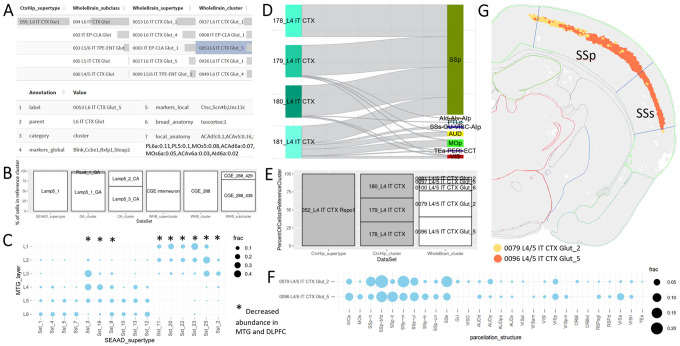
Addressing biological questions through annotation comparison in the healthy adult brain. **A)** Translation of mouse cortical cell types between studies. Top: Bar plot show annotations of 059_L6 IT CTX Osr1 cells from a study of mouse isocortex and hippocampus (Yao et al 2021) in a newer study of whole mouse brain (Yao et al 2023). Bottom: Cell type annotations for 0053 L6 IT CTX Glut_5 include information about cell type markers and anatomical localization. **B**) Translation of an interneuron cell type (Lamp5_1) across several studies of adult human neocortex or whole brain. **C**) Confusion matrix comparing dissected cortical layer and mapped SEA-AD supertypes (from Figure 3B) for Sst cell from Hodge, Bakken, et al 2019. Dots represent fractions of cells from a cortical layer mapping to a specific SEAAD_supertype (rows sum to 1). Supertypes showing decreased abundance in MTG and DLPFC in Gabitto, Travaglini et al 2024 (*) were preferentially located in superficial layers. **D-G**) Identification of neurons somatosensory (SS)-specific neuronal cell types. **D**) River plot showing the cluster assignment (left) and anatomic dissection (right) for cells in the only supertype primarily including cells from SSp (052_L4 IT CTX Rspo1). **E**) Bar plot showing the relationship between clusters in whole mouse brain and the three SSp-specific clusters in mouse isocortex plus hippocampus identified in **D**. **F**) Spatial localization in whole mouse brain (x-axis) for the two dominant whole mouse brain clusters identified in **E** (y-axis). Dots scaled as in **C** (rows sum to 1). **G**) A single hemisphere of MERFISH tissue section C57BL6J-63885.43 from the Allen Brain Cell Atlas (RRID:SCR_024440) shows that all cells from these two clusters are highly localized to layer 4 in primary (SSp) and secondary (SSs) somatosensory cortex.

By selecting the appropriate data set and exploring individual annotations for the cell type of interest, we see that “059_L6 IT CTX Osr1” includes cells from multiple cell types in the whole mouse brain (Figure 4A), suggesting that L6 IT cells are subdivided differently in both taxonomies and that there is higher cell type resolution in the whole mouse brain taxonomy. Most of the names include the term “L6 IT” suggesting that a consistent broader cell type resolution is retained. Finally, by selecting a particular whole brain cluster, we can identify relevant marker genes and we see that these cells come from multiple neocortical areas, suggesting this cell type is broadly found across neocortex and not restricted to a single brain region. Similar questions can be asked when linking other data sets, including human studies focused on MTG and the whole brain (Figure 4B).

#### How do we know that somatostatin interneurons impacted in AD are primarily found in superficial cortical layers?

As mentioned above, only a subset of SST types show decreased abundance in AD (Figure 3A). We reported previously that these cell types are primarily found in layers 2 and 3 of the human neocortex (Gabitto *et al*., 2024), but how can we use ACE to confirm? Our initial MTG study included laminar dissections, which are included in ACE. In Use Case #1, we mapped cells from this study to SEA-AD using MapMyCells (Figure 2B) and we have shared this as a precomputed table in ACE. We find that nearly all SST cells collected from cortical layer 1, 2, and 3 and some SST cells from layer 4 mapped to cell types showing decreased abundance in AD (Figure 4C), as reported.

#### Are area-specific isocortical cell types also found in subcortical structures and/or spatially organized?

Primary sensory cortices are highly specialized in mammals, with humans having a greatly expanded visual cortex and mice having specialized barrel fields in primary somatosensory cortex (SSp). In humans, several specialized neuronal types are found in VISp that are primarily localized to layer 4 (Jorstad, Close, *et al*., 2023). Using ACE we assessed whether mice showed similarly specialized cell types in SSp. To do this we first plotted a confusion matrix comparing the cell types in mouse cortex and hippocampus with the dissections from which they were collected, identifying a single supertype, 052_L4 IT CTX Rspo1, predominantly found in SSp (Supplemental Figure 2). This supertype includes four clusters, three of which are almost exclusive to SSp, and one with mixed expression across other largely primary sensory areas (Figure 4D). We then filtered our analysis to only include cells in these three SSp-specific, finding >75% of these cells were reassigned to two clusters in the whole mouse brain (Figure 4E).

We next shifted annotation tables in ACE to one focused on spatial localization of brain cell types and filtered cells to include only those found in 0079 L4/5 IT CTX Glut_2 or 0096 L4/5 IT CTX Glut_5. Consistent with matched clusters from mouse cortex plus hippocampus the majority of cells in these clusters were found in primary (SSp) or secondary (SSs) somatosensory cortex, although the distribution of these two clusters across subcompartments appeared slightly different (Figure 4F). To identify the precise spatial localization of cells from these two clusters in the whole mouse brain, we viewed a mouse MERFISH data set with reconstructed spatial coordinates using the Allen Brain Cell Atlas. These two clusters were highly spatially localized in layer 4 of isocortex, extending just beyond the reported boundaries of SSp and SSs, with cells in 0079 L4/5 IT CTX Glut_2 tending to be found more dorsally than cells in 0096 L4/5 IT CTX Glut_5 (Figure 4G). Out of the ~35,000 cells in these two clusters, ~70% were in SSp, ~15% in SSs, and only five were found outside isocortex (likely representing errors in label transfer). To assess potential functional relevance of these cell types in somatosensory cortex we used ACE to explore reported marker genes for these clusters (Yao *et al*., 2023). Interestingly, a conditional knockout in rhombomeres 3–5 of the top local marker gene for 0079 L4/5 IT CTX Glut_2 (*Robo3*) has been reported to induce bilateral innervation of the somatosensory thalamus and cortex, resulting in a barrel field of smaller, duplicated barrels representing both functional contra- and ipsilateral sensory inputs, with a decrease in morphological complexity of layer 4 spiny stellate cells within barrels (Tsytsarev *et al*., 2022). Thus, the distinct localization of these SSp-selective cell types likely has structural and functional consequences when key genes are disrupted.

These use cases represent common examples of how ACE can be used in cell type classification and comparison across studies; however, the visualizations included are highly generic. As a proof of principle of the versatility of ACE we present two additional use cases focused on disease diagnostics and treatment and on exploration of US Census data for interested readers (Supplemental Text).

## Discussion

ACE is an interactive tool that helps researchers compare cell type assignments and other metadata across different datasets. It allows users to upload their own data or choose from several predefined datasets, filter the data to focus on specific data points of interest, and then explore the relationships between the annotations using a variety of interactive visualizations. For example, ACE can show how the classification of a particular cell type changes between two different studies, how user-provided clusters relate to MapMyCells mapping results, or how the abundance of a cell type changes across different disease states. Overall, ACE is a versatile tool that can be used to explore and compare a wide variety of data annotations.

ACE is, to our knowledge, unique in its focus on cell annotations and metadata, its combination of user-provided and public data, and its ease of use. While other tools like CZI CellXGene (Megill *et al*., 2021), Allen Brain Cell Atlas (RRID:SCR_024440), Cytosplore (Abdelaal *et al*., 2020), and Cirrocumulus (Li *et al*., 2020) visualize and analyze single-cell (or spatial) transcriptomics data, displaying cells in 2D and allowing color-coding by metadata, their main purpose is gene expression analysis. Some of these tools also offer features like differential expression calculations and latent space regeneration, and can link cell type knowledge across multiple studies and organ systems. However, they are limited by their focus on a specific data type and often involve very large files (several gigabytes or more) that can be difficult to work with. Although many of ACE’s functions could be replicated with R or Python code, doing so requires moderate coding skills, restricting access for many users. ACE, requiring only simple text files and a web browser, is easily accessible to researchers in any field. Finally, the pre-built annotation files for Allen Institute cell type taxonomies address common requests from both the Allen Institute and the broader neuroscience community, allowing ACE to fill a crucial gap in the web tools available on Allen Brain Map. At the same time, ACE can be applied to a broad range of use cases, from cell type characterization to exploration of US Census data.

ACE has some limitations and opportunities for future improvement. First, since it is a lightweight tool written in R shiny and hosted on shinyapps.io, there are some constraints on file size which requires subsampling large data sets for upload and to optimize performance. This could lead to potential biases in statistical analysis that we have aimed to mitigate by evenly subsampling by cluster. Second, the ACE code base has been developed over several years to target an evolving set of use cases and uses some out of data R libraries and file formats. This is currently addressed by using a fixed R environment and by imposing constraints on the cell annotation table, but future efforts are needed to relax these constraints. Third, the cell typing field is ever evolving, and therefore ACE will never contain all relevant brain studies in adult brain and AD, without even considering other diseases or brain development. The primary way ACE addresses this limitation is by allowing upload of user data. In addition, we plan to periodically add additional prepopulated annotation tables to match cell type taxonomies included on Allen Brain Map in MapMyCells and the Allen Brain Cell Atlas to facilitate cross-platform data analysis. Finally, while we’ve attempted to anticipate the features that would be of use to the general neuroscience community, there are still a few outstanding issues to address that we know about (e.g., addition of bookmarking, data downloads, and statistics). We plan to continue improving ACE functionality and would encourage anyone that uses the tool to provide feedback on what would be of most value.

## Conclusions

Annotation Comparison Explorer combines standard and custom visualizations into a user-friendly, open-source web tool for exploring categorical and numeric relationships. In particular, ACE can translate cell type classifications and knowledge across studies of health and disease and can relate these annotations to underlying cell metadata such as spatial localization, donor information, and quality control metrics. Please include ACE in your cell type classification studies and provide feedback or contribute to this tool.

## Methods

### Data and code availability

ACE is available as a hosted website at https://sea-ad.shinyapps.io/ACEapp/ with source code and associate annotation tables available on GitHub at https://github.com/AllenInstitute/ACE. Although ACE is under active development, a snapshot of the project associated with this manuscript release is stored on Zenodo at https://doi.org/10.5281/zenodo.14624012 and on GitHub.

### Creation of a hosted R shiny application

ACE was created using R shiny (Chang *et al*., 2023), an extension of the R programming language supporting interactive analysis and visualization, and then uploaded to shinyapps.io so it is accessible anywhere on the web without the need for users to install on their computer or use R. ACE can read annotation tables from several file formats including h5ad, feather, and most commonly text files (csv and csv.gz) which can be quickly uploaded using vroom (Hester *et al*., 2023). Visualizations were created using a combination of custom functions and functions available as part of the ggplot2 (Wickham, 2016) and rbokeh (Hafen and Continuum Analytics, Inc., 2021) R packages. The R package renv (Ushey and Wickham, 2023) is used to manage project-specific libraries and dependencies, ensuring reproducibility between shinyapps.io and local environments. Web app usage is being tracked using Google Analytics; no user information (beyond city of origin) or uploaded data is retained for any purpose. A video webinar and detailed written user guide have been created to improve web application usability.

### Generating annotation tables for cell type classification in healthy adult brain

ACE includes several predefined annotation tables comparing cell type classifications across public studies of mouse and human brain. Each table (or “dataset”) includes a description describing the specific taxonomies and annotations included therein. All tables except the ones noted below were generated by (1) identifying a set of studies that were performed using an overlapping set of cells, (2) subsetting existing cell annotation tables from each study to only include these common cells, and (3) compiling relevant cell annotations (typically cell type assignments) from each study into a single table. Large data sets were subsampled to less than ~500,000 cells to speed up visualizations.

The “Middle temporal gyrus (MTG) (initial studies)” table connected taxonomies from two distinct studies of MTG by using MapMyCells (RRID:SCR_024672; https://portal.brain-map.org/atlases-and-data/bkp/mapmycells) to transfer labels from the recent human MTG SEA-AD taxonomy (Gabitto *et al*., 2024) to cells in an earlier MTG taxonomy (Hodge *et al*., 2019) that has been used as a reference for several human patch-seq studies. Both the original and transferred labels, along with some additional cell metadata, are included in the annotation table. The same process was also used to map cells from SEA-AD to the whole human brain taxonomy (Siletti *et al*., 2023) for the “Middle temporal gyrus (recent studies)” although all other annotations were done by matching cells (as above). Annotation tables from two patch-seq studies are included as well (Gouwens *et al*., 2020; Scala *et al*., 2021). In both cases, multiple cell type classifications were defined in the original publications and are reproduced in ACE for visualization.

Numeric values from some studies are reproduced in ACE, including UMAP coordinates, physical spatial locations and cell type assignment confidence scores (for “Spatial localization of brain cell types”), and electrophysiological features (for patch-seq studies). Mapping confidences calculated by MapMyCells are also provided for the initial MTG studies.

### Generating annotation tables comparing cell types across studies of Alzheimer’s disease

To link annotations across 11 studies of AD, we integrated data from ten community-based studies of AD focused on DFC (Mathys *et al*., 2023, 2019; Olah *et al*., 2020; Zhou *et al*., 2020; Lau *et al*., 2020; Leng *et al*., 2021; Morabito *et al*., 2021; Yang *et al*., 2022; Cain *et al*., 2023; Green *et al*., 2023) with DFC data from SEA-AD (Gabitto *et al*., 2024) and then compared transferred SEA-AD labels with published information for each study. Integration and label transfer was performed as described in (Gabitto *et al*., 2024). Briefly, raw sequencing data was downloaded from the AD Knowledge Portal hosted on Synapse or from the Sequencing Read Archive (SRA) and processed using the same pipeline as was used for SEA-AD snRNA-seq data. Processed data were similarly QCed and then mapped to SEA-AD cell types using a variation of the Deep Generative Mapping model on MapMyCells. Cell type classifications from the original studies were collected from Synapse, original manuscripts, and email correspondence with authors and linked to mapped cell type using unique cell identifiers, only retaining cells with both SEA-AD and initial cell type assignments for additional processing.

Since ACE relies on common mappings to the same cell, each community study was linked to a subsampled set of SEA-AD cells probabilistically as part of the SEA-AD annotation table. For each study, a confusion matrix comparing mapped SEA-AD cell types (supertypes) with the highest resolution cell type reported was generated to calculate the fraction of cells from each SEA-AD supertypes coming from each assigned cell type in that study. Each cell was then assigned a cell type probabilistically based on the fractions corresponding to the original SEA-AD type. This process was then repeated for each of the 10 studies. Cells with SEA-AD supertypes that were not found in a given study were assigned a cell type of “noMappedCells”. This occurred most frequently when comparing neuronal cells to studies focused on glial cell types. Cell type assignments for broader cell type definitions were inferred from their higher-resolution counterparts.

Cell types from all 11 studies were also assigned a direction change in abundance with AD (up, down, or unchanged). These assignments were made by reviewing the published manuscripts for quantitative values presented in the text or in figures (when available) or for qualitative statements (when values were not presented) and then were recorded in the cell type annotation table. In some cases we recorded notes in this table indicating how direction changes were defined.

### Figure creation

All figure panels except 2A and 4G were created using the web version of ACE, either by taking screenshots or by downloading images using the download buttons therein. While some minor rearrangements and additions of supporting information were made for clarity, no edits were made to the data shown in any visualization. Figure panel 2A was created in PowerPoint. Figure panel 4G was taken as a screenshot from the Allen Brain Cell atlas, and can be reconstructed using the following URL: https://knowledge.brain-map.org/abcatlas#AQEBSzlKTjIzUDI0S1FDR0s5VTc1QQACSFNZWlBaVzE2NjlVODIxQldZUAADBAFGUzAwRFhWMFQ5UjFYOUZKNFFFAAIAAAFRWTVTOEtNTzVITEpVRjBQMDBLAAIAAAExNUJLNDdEQ0lPRjFTTExVVzlQAAIAAAFDQkdDMFUzMFZWOUpQUjYwVEpVAAICMDA3OSBMNC81IElUIENUWCBHbHV0XzIAMDA5NiBMNC81IElUIENUWCBHbHV0XzUAAAQBAAKEnp2fgjSU5gOCKpijglxyWgQyTlFUSUU3VEFNUDhQUUFITzRQAAWBr6ZKgemsDoGggUeAktXoBgAHAAAFAQFTY24zYgAABgEBAkNCR0MwVTMwVlY5SlBSNjBUSlUAA34AAAAEAAAIVkZPRllQRlFHUktVRFFVWjNGRgAJTFZEQkpBVzhCSTVZU1MxUVVCRwAKAAsBVExPS1dDTDk1UlUwM0Q5UEVURwACNzNHVlREWERFR0UyN00yWEpNVAADAQQBAAIjMDAwMDAwAAPIAQAFAQECIzAwMDAwMAADyAEAAAACAQA%3D.

## Supplementary Material

Supplement 1

Supplement 2

## Figures and Tables

**Table 1:** List of transcriptomics atlases on Allen Brain Map and their associated tools. An extended version available on Allen Brain Map at https://portal.brain-map.org/help-and-community/guide-cell-types. Abbreviations: VISp, primary visual cortex; ALM, anterior lateral motor cortex; MTG, middle temporal gyrus; M1 and MOp, primary motor cortex. Notes: **(1)**, The data exploration tool presents a draft version of the “cross-areal” taxonomy, which does not match the one in the manuscript or in ACE; **(2)** This study has two instances of Transcriptomics Explorer; only one is shown; **(3)** “M1 – 10x genomics (2020)” is a subset of the data from this study; **(4)** Only a subset of SEA-AD tools are presented here. See sea-ad.org for additional information; **(5)** Two separate studies are included in the ABC Atlas that use the same taxonomy generated from Yao et al 2023; **(6)** Data from this study also available on GitHub and at CELLxGENE. EEL FISH is also used in this study but is not available on Allen Brain Map. All tools in this table can be accessed from brain-map.org under the “Cell Types” tab.

Name on Allen Brain Map	Reference	Omics technique(s)	Brain region(s) targeted	Species	Data exploration tool(s)	Note
V1 & ALM - SMART-seq (2018)	Tasic *et al*. 2018	SMART-Seq v4	VISp; ALM	Mouse	RNA-Seq Data Navigator: Mouse	
MTG - SMART-seq (2018)	Hodge *et al*. 2019	SMART-Seq v4	MTG	Human	RNA-Seq Data Navigator: Human	
Multiple Cortical Areas - SMART-seq (2019)	Jorstad, Close, *et al*. 2023	SMART-Seq v4; 10xv2	Eight neocortical brain regions (including MTG and M1)	Human	Transcriptomics Explorer	1
Whole Cortex & Hippocampus (2019/2020)	Yao, van Velthoven, *et al*. 2021	SMART-Seq v4; 10xv3	Isocortex and hippocampal formation	Mouse	Transcriptomics Explorer; Taxonomy browser tool	2
M1 - 10x genomics (2020)	Bakken *et al*. 2021	10xv3	M1	Human	Transcriptomics Explorer	
Cell Type Knowledge Explorer	Yao, Liu, *et al*. 2021; Bakken *et al*. 2021	SMART-Seq v4; 10xv3	M1; MOp	Human; Mouse; Marmoset	Cell Type Knowledge Explorer	3
Seattle Alzheimer’s Disease Brain Cell Atlas (SEA-AD)	Gabitto *et al*. 2024	10xv3; MERFISH	MTG	Human	Transcriptomics Explorer; Allen Brain Cell Atlas; MapMyCells	4
Mouse whole-brain transcriptomic cell type atlas	Yao *et al*. 2023; Zhang *et al*. 2023	10xv2; 10xv3; MERFISH	Whole brain	Mouse	Allen Brain Cell Atlas; MapMyCells	5
Transcriptomic diversity of cell types in adult human brain	Siletti *et al*. 2023	10xv3	Whole brain	Human	Allen Brain Cell Atlas; MapMyCells	6

## References

[R1] Abdelaal T. (2020) Cytosplore-Transcriptomics: a scalable inter-active framework for single-cell RNA sequencing data analysis. 2020.12.11.421883.

[R2] Bakken T.E. (2021) Comparative cellular analysis of motor cortex in human, marmoset and mouse. Nature, 598, 111–119. 34616062 10.1038/s41586-021-03465-8 PMC8494640

[R3] Berg J. (2021) Human neocortical expansion involves glutamatergic neuron diversification. Nature, 598, 151–158. 34616067 10.1038/s41586-021-03813-8 PMC8494638

[R4] Cain A. (2023) Multicellular communities are perturbed in the aging human brain and Alzheimer’s disease. Nat Neurosci, 26, 1267–1280. 37336975 10.1038/s41593-023-01356-x PMC10789499

[R5] Chang W. (2023) shiny: Web Application Framework for R.

[R6] Chartrand T. (2023) Morphoelectric and transcriptomic divergence of the layer 1 interneuron repertoire in human versus mouse neocortex. Science, 382, eadf0805. 10.1126/science.adf0805 PMC11864503 37824667

[R7] Deczkowska A. (2018) Disease-Associated Microglia: A Universal Immune Sensor of Neurodegeneration. Cell, 173, 1073–1081. 29775591 10.1016/j.cell.2018.05.003

[R8] Gabitto M.I. (2024) Integrated multimodal cell atlas of Alzheimer’s disease. 2023.05.08.539485. 10.1038/s41593-024-01774-5 PMC11614693 39402379

[R9] Gouwens N.W. (2020) Integrated Morphoelectric and Transcriptomic Classification of Cortical GABAergic Cells. Cell, 183, 935–953.e19. 33186530 10.1016/j.cell.2020.09.057 PMC7781065

[R10] Green G.S. (2023) Cellular dynamics across aged human brains uncover a multicellular cascade leading to Alzheimer’s disease Neuroscience.

[R11] Grindberg R.V. (2013) RNA-sequencing from single nuclei. Proceedings of the National Academy of Sciences of the United States of America, 110, 19802–7. 24248345 10.1073/pnas.1319700110 PMC3856806

[R12] Hafen R. and Continuum Analytics, Inc. (2021) rbokeh: R Interface for Bokeh.

[R13] Hao Y. (2021) Integrated analysis of multimodal single-cell data. Cell, 184, 3573–3587.e29. 34062119 10.1016/j.cell.2021.04.048 PMC8238499

[R14] Hester J. (2023) vroom: Read and Write Rectangular Text Data Quickly.

[R15] Hodge R.D. (2019) Conserved cell types with divergent features in human versus mouse cortex. Nature, 573, 61–68. 31435019 10.1038/s41586-019-1506-7 PMC6919571

[R16] Jorstad N.L., Song J.H.T., (2023) Comparative transcriptomics reveals human-specific cortical features. Science, 382, eade9516. 10.1126/science.ade9516 PMC10659116 37824638

[R17] Jorstad N.L., Close J., (2023) Transcriptomic cytoarchitecture reveals principles of human neocortex organization. Science, 382, eadf6812. 10.1126/science.adf6812 PMC11687949 37824655

[R18] Kalmbach B.E. (2021) Signature morpho-electric, transcriptomic, and dendritic properties of human layer 5 neocortical pyramidal neurons. Neuron, 109, 2914–2927.e5. 34534454 10.1016/j.neuron.2021.08.030 PMC8570452

[R19] Keren-Shaul H. (2017) A Unique Microglia Type Associated with Restricting Development of Alzheimer’s Disease. Cell, 169, 1276–1290.e17. 28602351 10.1016/j.cell.2017.05.018

[R20] Lau S.-F. (2020) Single-nucleus transcriptome analysis reveals dysregulation of angiogenic endothelial cells and neuroprotective glia in Alzheimer’s disease. Proc. Natl. Acad. Sci. U.S.A., 117, 25800–25809. 32989152 10.1073/pnas.2008762117 PMC7568283

[R21] Lee B.R. (2023) Signature morphoelectric properties of diverse GABAergic interneurons in the human neocortex. Science, 382, eadf6484. 10.1126/science.adf6484 PMC12229143 37824669

[R22] Leng K. (2021) Molecular characterization of selectively vulnerable neurons in Alzheimer’s disease. Nature Neuroscience, 24, 276–287. 33432193 10.1038/s41593-020-00764-7 PMC7854528

[R23] Li B. (2020) Cumulus provides cloud-based data analysis for large-scale single-cell and single-nucleus RNA-seq. Nat Methods, 17, 793–798. 32719530 10.1038/s41592-020-0905-x PMC7437817

[R24] Mathys H. (2023) Single-cell atlas reveals correlates of high cognitive function, dementia, and resilience to Alzheimer’s disease pathology. Cell, 186, 4365–4385.e27. 37774677 10.1016/j.cell.2023.08.039 PMC10601493

[R25] Mathys H. (2019) Single-cell transcriptomic analysis of Alzheimer’s disease. Nature, 570, 332–337. 31042697 10.1038/s41586-019-1195-2 PMC6865822

[R26] Megill C. (2021) cellxgene: a performant, scalable exploration platform for high dimensional sparse matrices. 2021.04.05.438318.

[R27] Miller J.A. (2020) Common cell type nomenclature for the mammalian brain. eLife, 9, e59928. 33372656 10.7554/eLife.59928 PMC7790494

[R28] Morabito S. (2021) Single-nucleus chromatin accessibility and transcriptomic characterization of Alzheimer’s disease. Nature Genetics, 53, 1143–1155. 34239132 10.1038/s41588-021-00894-z PMC8766217

[R29] Olah M. (2020) Single cell RNA sequencing of human microglia uncovers a subset associated with Alzheimer’s disease. Nat Commun, 11, 6129. 33257666 10.1038/s41467-020-19737-2 PMC7704703

[R30] Patil P. California Independent Medical Review Dataset.

[R31] Regev A. (2017) The Human Cell Atlas. eLife, 6, e27041. 29206104 10.7554/eLife.27041 PMC5762154

[R32] Scala F. (2021) Phenotypic variation of transcriptomic cell types in mouse motor cortex. Nature, 598, 144–150. 33184512 10.1038/s41586-020-2907-3 PMC8113357

[R33] Sikkema L. (2023) An integrated cell atlas of the lung in health and disease. Nat Med, 29, 1563–1577. 37291214 10.1038/s41591-023-02327-2 PMC10287567

[R34] Siletti K. (2023) Transcriptomic diversity of cell types across the adult human brain. Science, 382, eadd7046. 10.1126/science.add7046 37824663

[R35] Tang F. (2009) mRNA-Seq whole-transcriptome analysis of a single cell. Nat Methods, 6, 377–382. 19349980 10.1038/nmeth.1315

[R36] Tasic B. (2018) Shared and distinct transcriptomic cell types across neocortical areas. Nature, 563, 72–78. 30382198 10.1038/s41586-018-0654-5 PMC6456269

[R37] Tsytsarev V. (2022) Layers 3 and 4 Neurons of the Bilateral Whisker-Barrel Cortex. Neuroscience, 494, 140–151. 35598701 10.1016/j.neuroscience.2022.05.018 PMC9884091

[R38] U.S. Department of Agriculture, Economic Research Service. County-Level Data Sets: Download Data.

[R39] Ushey K. and Wickham H. (2023) renv: Project Environments.

[R40] Wickham H. (2016) ggplot2: Elegant Graphics for Data Analysis Springer-Verlag New York.

[R41] Yang A.C. (2022) A human brain vascular atlas reveals diverse mediators of Alzheimer’s risk. Nature, 603, 885–892. 35165441 10.1038/s41586-021-04369-3 PMC9635042

[R42] Yao Z. (2023) A high-resolution transcriptomic and spatial atlas of cell types in the whole mouse brain. Nature, 624, 317–332. 38092916 10.1038/s41586-023-06812-z PMC10719114

[R43] Yao Z., van Velthoven C.T.J., (2021) A taxonomy of transcriptomic cell types across the isocortex and hippocampal formation. Cell, 184, 3222–3241.e26. 34004146 10.1016/j.cell.2021.04.021 PMC8195859

[R44] Yao Z., Liu H., (2021) A transcriptomic and epigenomic cell atlas of the mouse primary motor cortex. Nature, 598, 103–110. 34616066 10.1038/s41586-021-03500-8 PMC8494649

[R45] Zhang M. (2023) Molecularly defined and spatially resolved cell atlas of the whole mouse brain. Nature, 624, 343–354. 38092912 10.1038/s41586-023-06808-9 PMC10719103

[R46] Zhou Y. (2020) Human and mouse single-nucleus transcriptomics reveal TREM2-dependent and TREM2-independent cellular responses in Alzheimer’s disease. Nature Medicine, 26, 131–142. 10.1038/s41591-019-0695-9 PMC6980793 31932797

