# Supplementary material for: Annotation Comparison Explorer (ACE): connecting brain cell types across studies of health and Alzheimer’s Disease
Source: bioRxiv. 2025 Feb 12:2025.02.11.637559. Preprint. [Version 1] doi: 10.1101/2025.02.11.637559 (PMC11844562; doi:10.1101/2025.02.11.637559)

Supplemental materials for:

Title: Annotation Comparison Explorer (ACE): connecting brain cell types across studies of health and Alzheimer's Disease

Author list: Jeremy A. Miller [1*], Kyle J. Travaglini [1], Tain Luquez [2], Rachel E. Hostetler [1], Aaron Oster [1], Scott Daniel [1], Bosiljka Tasic [1], Vilas Menon [2]
1. Allen Institute, 2. Columbia University, * Corresponding author

Contents:

Supplemental Text
Supplemental Tables 1-2
Supplemental Figure 1-4

Supplemental Text

This section includes two additional use cases for Annotation Comparison Explorer (ACE) outside of the scope of cell type annotation, presented as a proof of principle of the versatility of ACE in comparing any data annotations that can be represented as words or numbers. We emphasize that these two use cases represent exploratory efforts at testing the utility of ACE and would need to be contextualized before any conclusions could be drawn.

Supplemental Use Case 1: Disease diagnosis and treatment

One potential utility for ACE is relating disease diagnoses, treatments, and outcomes for different demographics. Here we downloaded information from the California Department of Managed Health Care (DMHC) provided as part of a prior kaggle competition (Patil) and uploaded it to ACE for exploration. While most diagnoses were relatively evenly distributed between males and females, “OB-Gyn/ Pregnancy” and “Prevention/Good Health” diagnoses were heavily skewed towards females (**Supplementary Figure 3A**), the latter at least partially due to preventive screenings for breast cancer (**Supplementary Figure 3B**). Similarly several diagnoses different by age with “Pediatrics” and “Mental” skewing young, while “Central Nervous System (CNS) / Neuromuscular” is found most commonly in the 51-64 year old group (**Supplementary Figure 3C**). Many of the mental subdiagnoses included neurodevelopmental disorders, while the CNS subdiagnoses included a mix of neurodegenerative disorders and other categories (**Supplementary Figure 3C-D**). Many diagnoses involved only a few treatments, with severe depression being treated with electrical interventions (e.g., brain stimulation), brain tumors requiring neurosurgery and cancer treatment, and paralysis requiring rehabilitation services and durable medical equipment, along with other connections that were less clear to a non-specialist.

Supplemental Use Case 2: Comparing demographic information across US counties

As another demonstration of the utility of ACE, we downloaded information about poverty, education, and unemployment rates and household income across counties in the United States collected as part of the U.S. Census (U.S. Department of Agriculture, Economic Research Service.), joining this information into a single table (**Supplementary Table 2**) for upload to ACE. Overall, metropolitan areas have higher median household incomes than non-metropolitan areas, even after accounting for high variability of income across states (**Supplementary Figure 4A**), with some of the wealthiest areas located near DC, Silicon Valley, and Los Alamos National Labs. We also found county-wide correlations between income and education (as measured by percent of adults with at least a bachelor’s education), poverty levels, and (to a lesser degree), unemployment rates (**Supplementary Figure 4B-D**), which all also show differences in metropolitan vs. non-metropolitan areas.

Supplemental Tables

Supplemental Table 1: Information each prebuilt annotation table available in ACE at the time of manuscript submission. All relevant data files are available at https://github.com/AllenInstitute/ACE/ in the data folder and on Zenodo (https://zenodo.org/records/14624012): *Note, this supplemental table spans three pages.*

**Annotation table name**	**Annotation table category**	**Description of annotation table and associated data sets**
Alzheimer's disease (SEA-AD vs. community)	Disease studies	Data and associated cell type assignments from ten studies of Alzheimer's disease. All data sets were mapped to SEA-AD data, and both the 10 data sets and the details of the data integration and mapping are described in Gabitto, Travaglini, et al 2023 (DOI:10.1101/2023.05.08.539485). For each data set, their mappings to SEA-AD as well as original cluster assignments are included in the tables. In addition, each cell type's change in abundance in AD from the original study, as well as some basic information about the cell types are included. Data is subsampled to 100 cells per SEA-AD supertype. The way data is encoded, comparisons between each data set an SEA-AD are valid, but comparisons CANNOT be accurately made between external data sets. If you have additional data sets you'd like to see included in this study, please reach out!
Spatial localization of brain cell types	Mouse cell type classification	[This data set may be slow to load--please be patient!] Data about brain cell types AND brain regions using MERFISH data collected from mouse whole brain (Yao et al 2023; DOI:10.1038/s41586-023-06812-z; AIT21). This includes cell type assignments and spatial positions for MERFISH data, subsampled to 50 cells per cluster + 5000 cells per section. Cells have been assigned to CCF parcellations to allow direct matching between cell types and anatomic structures. The current version of this table may have rotated CCF coordinates, making visualization challenging using the scatterplot view--new coordinates are in process, but this will not impact any other exploration components.

Middle temporal gyrus (recent studies)	Human cell type classification	Human MTG data set comparisons (recent). Cluster and other assignments for the SAME set of cells collected from MTG that were used in four published studies, including in SEA-AD and the whole human brain. These studies are (1) the human 'great ape' (GA) study (Jorstad, Song, Exposito-Alonso, et al 2023, DOI:10.1126/science.ade9516), which assesses cell types in MTG across species; (2) the human 'cross-areal' (CA) study (Jorstad et al 2023, DOI:10.1126/science.adf6812), which assesses cell types across multiple neocortical areas in human (including MTG); (3) 'SEA-AD' calls for each level of the taxonomy in MTG (Gabitto, Travaglini, et al 2023; DOI:10.1101/2023.05.08.539485)' and (4) a draft atlas of all cell types in the human brain (Siletti et al 2023; DOI:10.1126/science.add7046). The latter two of these studies are currently available on both MapMyCells and the ABC Atlas. Note: the clusters in the GA study represented the starting point for analysis in generation of the SEA-AD supertypes. All three MTG-focused data sets in this table were generated using a nearly identical set of cells collected using 10X genomics single nucleus RNA-seq technologies, and only the matching subset of cells from the whole human brain study are included. Clusters from the GA and CA studies are appended with '_GA' and '_CA', respectively, for ACE functionality.
Middle temporal gyrus (initial studies)	Human cell type classification	Human MTG data set comparisons (historical). Cluster assignments from the original study of cell types in human MTG from the Allen Institute ('Hodge', Bakken, et al 2019: DOI:10.1038/s41586-019-1506-7) alongside SEA-AD calls for each level of the taxonomy in MTG from Gabitto, Travaglini, et al 2023 (DOI:10.1101/2023.05.08.539485). Cortical layers of the initial dissections are also included per cell to allow for cell type by layer comparisons. We also include some example UMAP coordinates for visualization of metadata. Labels were transferred from the SEA-AD taxonomy to Hodge et al data using MapMyCells with deep generative model in October 2024.
Cortex + Hippocampus vs. whole brain	Mouse cell type classification	Data about brain cell types from mouse whole brain (Yao et al 2023; DOI:10.1038/s41586-023-06812-z; AIT21) and their mapping to mouse cortex + hippocampus (Yao et al 2021, DOI:10.1038/s41586-023-06812-z). This includes data from AIT21 downsampled to only include 100 cells per cluster from cortex + hippocampus. Any clusters from the whole brain that are NOT listed are either rare or absent in mouse cortex + hippocampus. We recommend using this table to translate forward (e.g., from the older to the newer taxonomy).
Initial cortex and hippocampus studies	Mouse cell type classification	Convert cell types from mouse primary visual (VISp), Anterior Lateral Motor area (ALM), and primary motor (MOp) cortex to mouse cortex + hippocampus. This is done using SmartSetV4 cells that were included in multiple studies and their associated cell type annotations: (1) VISp and ALM cells from Tasic et al 2018 (DOI:10.1038/s41586-018-0654-5), (2) MOp cells used in the Cell Type Knowledge Explorer (Yao, Liu, Xie, Fischer, et al 2021, DOI:10.1038/s41586-021-03500-8), and overlapping cells from mouse cortex + hippocampus (Yao et al 2023; DOI:10.1038/s41586-023-06812-z; AIT21). No subsampling was done for this table. Any clusters from mouse Ctx + hipp that are NOT listed are either rare or absent in mouse VISp and mouse MOp. We recommend using this table to translate forward (e.g., from the older to the newer taxonomy). Finally, note that MOp and VISp/ALM cannot be directly compared because different cells were included in these two studies.

Motor cortex vs. whole brain	Mouse cell type classification	Convert mouse cell type assignments from primary motor cortex (MOp) (Yao, Liu, Xie, Fischer, et al 2021, DOI:10.1038/s41586-021-03500-8) to whole mouse brain (Yao et al 2023; AIT21, DOI:10.1038/s41586-023-06812-z). This is done using 10X v2 and v3 cells that were included in both studies, but with no subsampling. Any clusters from mouse whole brain that are NOT listed are either rare or absent in mouse MOp. We recommend using this table to translate forward (e.g., from the older to the newer taxonomy).
Mouse visual cortex (GABAergic)	Patch-seq (shape + function + genes)	Mouse Patch-seq data from primary visual cortex (VISp). This table compares matched transcriptomic-type (T-Type) and morphoelectric transcriptomic type (MET-type) cell type assignments for the same cells from Gouwens, Sorensen, et al 2020 (DOI:10.1016/j.cell.2020.09.057). The cell type annotations for MET types was taken directly from supplemental materials in the manuscript. T-types were assigned through comparison with Tasic et al 2018 (DOI:10.1038/s41586-018-0654-5). In addition to cell type assignments, cell metadata and electrophysiological properties for each patch-seq cell is included for interactive visualization of electrophysiological properties.
Mouse motor cortex	Patch-seq (shape + function + genes)	Mouse Patch-seq data from primary motor cortex (MOp). Data from Scala, Kobak, et al 2020 (DOI:10.1038/s41586-020-2907-3). This table include transcriptomic types (T-Types) defined through comparison with MOp cell types linked in the Cell Type Knowledge Explorer (Yao, Liu, Xie, Fischer, et al 2021, DOI:10.1038/s41586-021-03500-8) and through comparison with primary visual cortex (VISp) types from Tasic et al 2018 (DOI:10.1038/s41586-018-0654-5). In addition to cell type assignments, cell metadata and electrophysiological properties for each patch-seq cell is included for interactive visualization of electrophysiological properties.

Supplemental Table 2: County level information collected from publicly accessible data at the USDA from https://www.ers.usda.gov/data-products/county-level-data-sets/county-level-data-sets-download-data, and compiled into a single file. The "TableOfCountyInfo" sheet includes the actual data uploaded to ACE in .csv format, while the "VariableDescription" describes each column in the first sheet using definitions from the website above.

This table was uploaded as a separate Supplemental File.

Supplemental Figures

Supplemental Figure 1: Small example to demonstrate how ACE works. A) Two example sets of annotations for the same set of eight cells; one before and one after a ninth cell is added. Brief definitions shown below. **B)** Encoding of this example from **A** into the cell table (top) and optional annotation table (bottom) required for input into ACE. **C)** Representation of a river plot showing the relationships between the two sets of annotations for the eight cells. Note that ACE will produce a river plot like this one if the tables from **B** are uploaded.

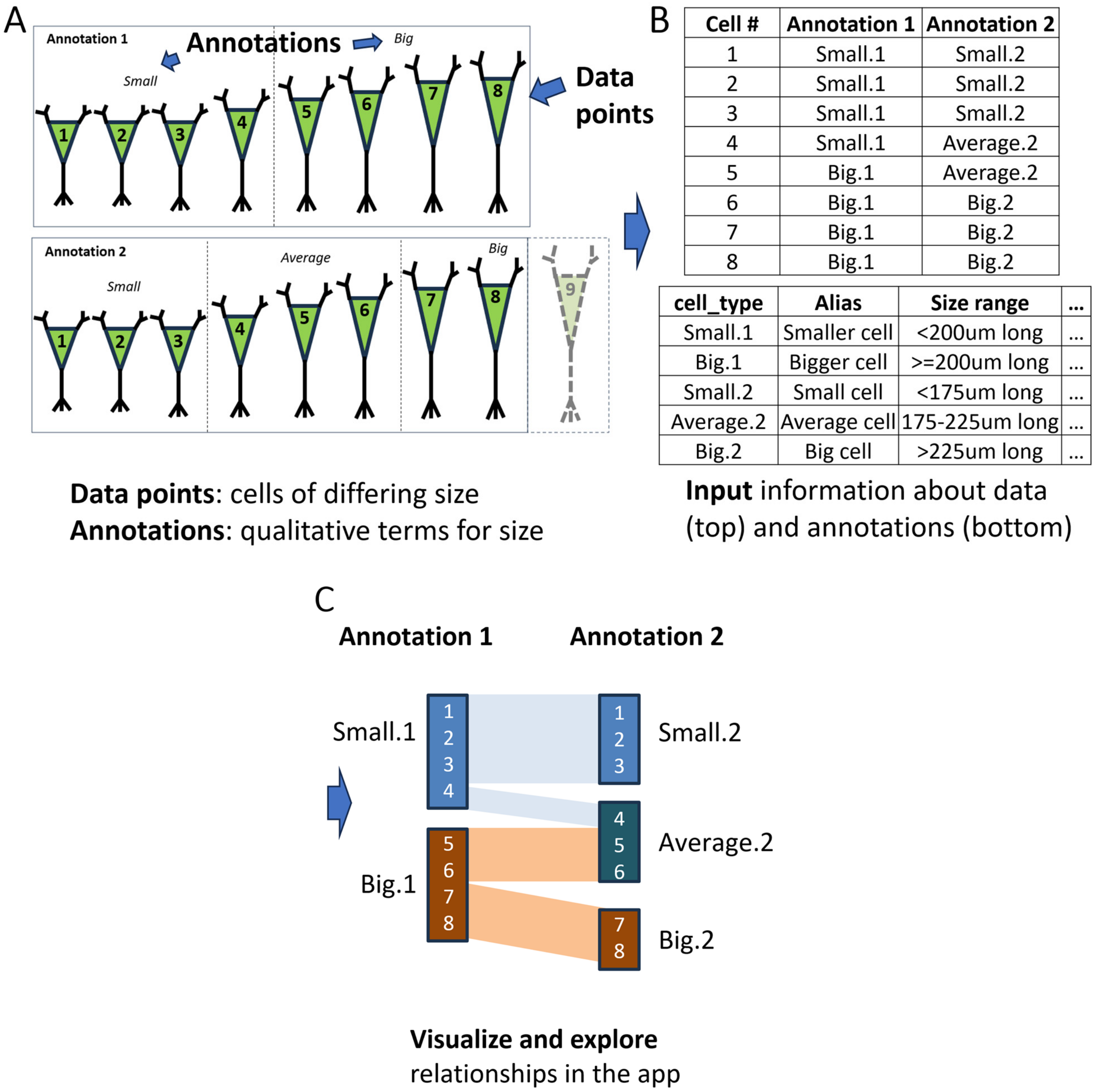

Cell #	Annotation 1	Annotation 2
1	Small.1	Small.2
2	Small.1	Small.2
3	Small.1	Small.2
4	Small.1	Average.2
5	Big.1	Average.2
6	Big.1	Big.2
7	Big.1	Big.2
8	Big.1	Big.2

cell_type	Alias	Size range	...
Small.1	Smaller cell	<200um long	...
Big.1	Bigger cell	>=200um long	...
Small.2	Small cell	<175um long	...
Average.2	Average cell	175-225um long	...
Big.2	Big cell	>225um long	...

Supplemental Figure 2: Spatial localization of cell types in mouse cortex and hippocampus. Confusion matrix showing the dissection structure (x-axis) of cells mapping to each isocortex plus hippocampus supertype (y-axis). Rows are ordered to maximize the diagonal and line up supertypes with similar spatial profiles. Dots scaled as in **Figure 4C** (rows sum to 1). Blue arrow highlights 0053 L6 IT CTX Glut_5, the only cell type primarily collected from SSp. *Note, this supplemental figure spans two pages.*

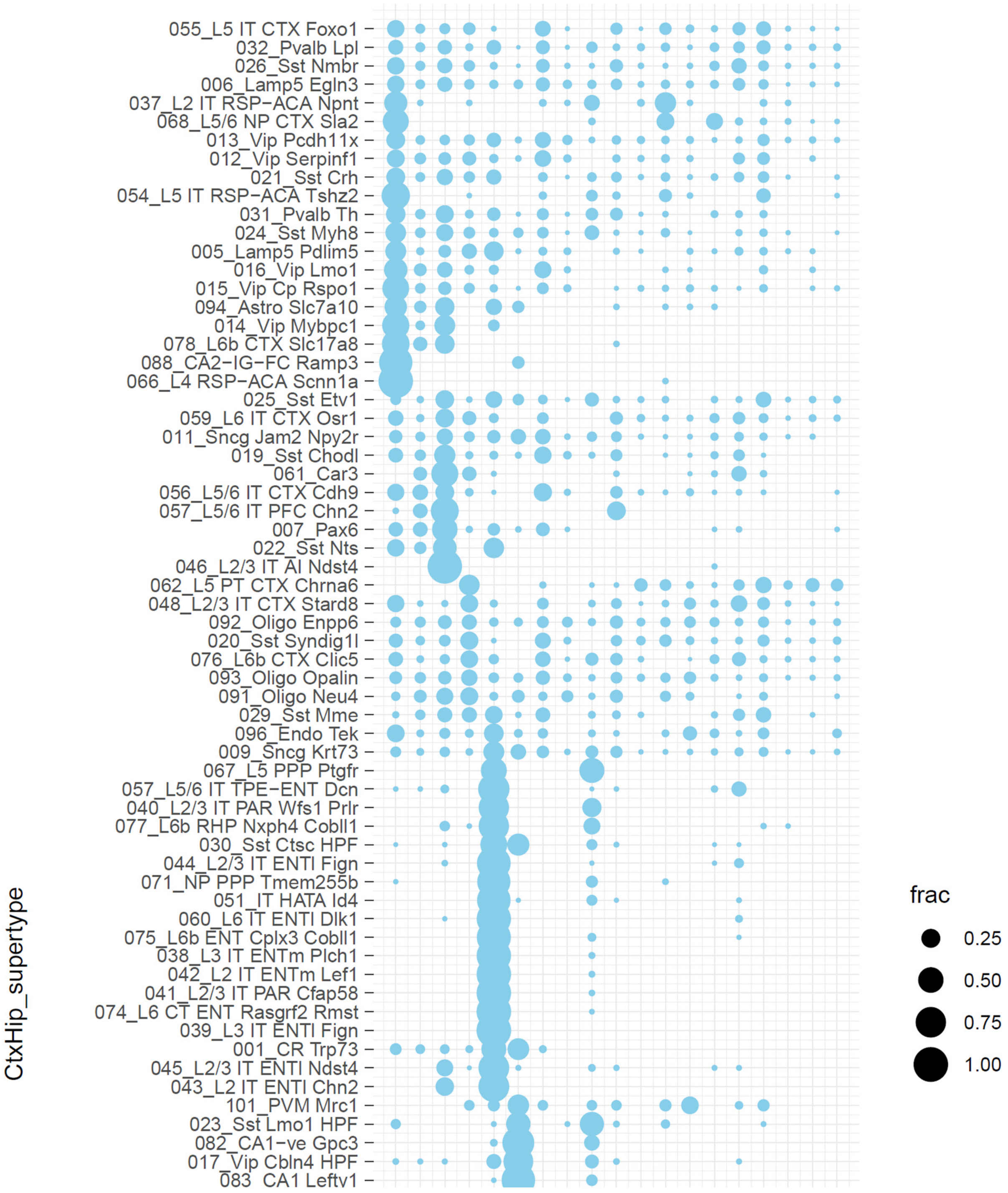

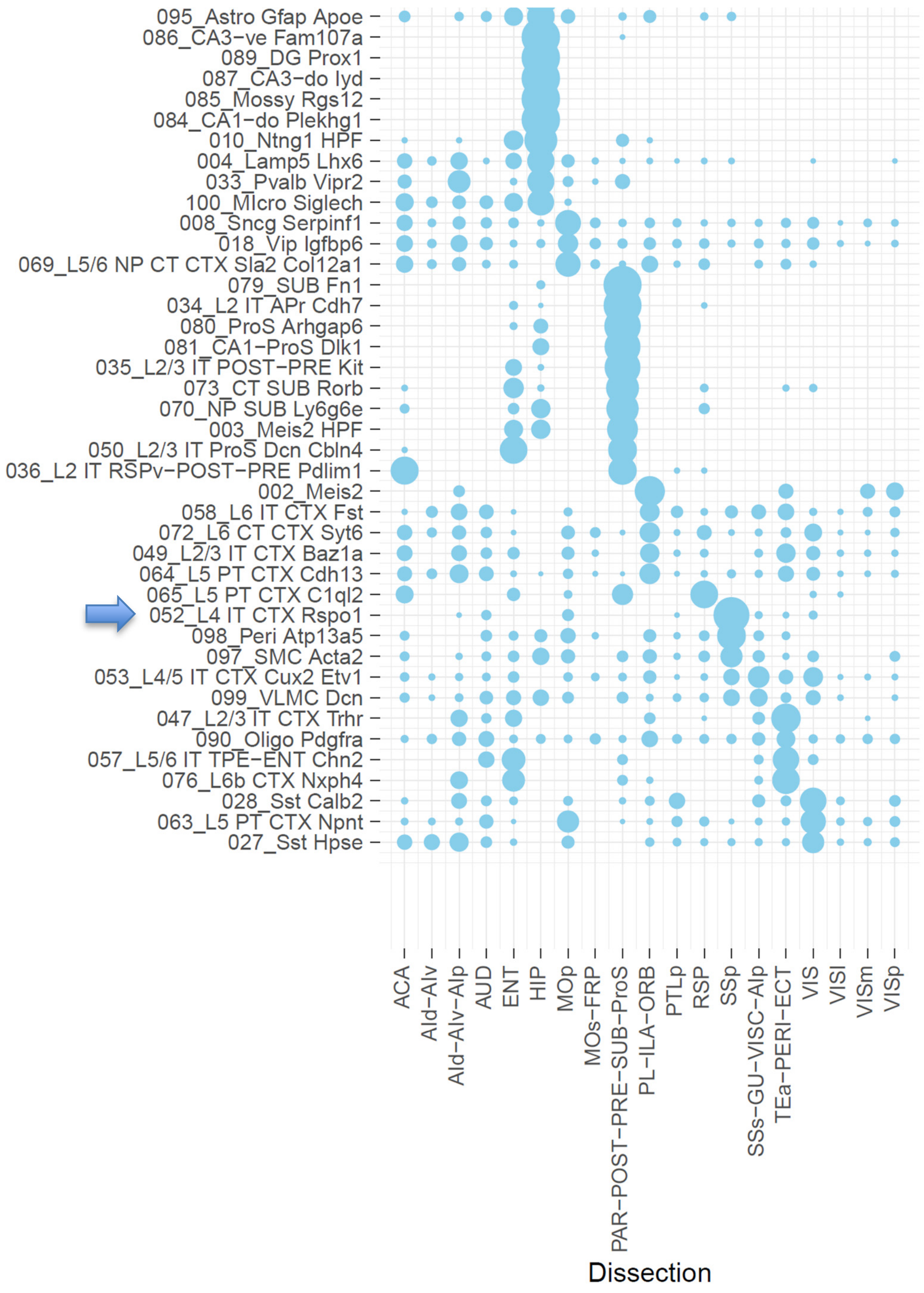
095_Astro Gfap Apoe
086_CA3−ve Fam107a
089_DG Prox1
087_CA3−do Iyd
085_Mossy Rgs12
084_CA1−do Plekhg1
010_Ntng1 HPF
004_Lamp5 Lhx6
033_Pvalb Vipr2
100_Micro Siglech
008_Sncg Serpinf1
018_Vip Igfbp6
069_L5/6 NP CT CTX Sla2 Col12a1
079_SUB Fn1
034_L2 IT APr Cdh7
080_ProS Arhgap6
081_CA1−ProS Dlk1
035_L2/3 IT POST−PRE Kit
073_CT SUB Rorb
070_NP SUB Ly6g6e
003_Meis2 HPF
050_L2/3 IT ProS Dcn Cbln4
036_L2 IT RSPv−POST−PRE Pdlim1
002_Meis2
058_L6 IT CTX Fst
072_L6 CT CTX Syt6
049_L2/3 IT CTX Baz1a
064_L5 PT CTX Cdh13
065_L5 PT CTX C1ql2
052_L4 IT CTX Rspo1
098_Peri Atp13a5
097_SMC Acta2
053_L4/5 IT CTX Cux2 Etv1
099_VLMC Dcn
047_L2/3 IT CTX Trhr
090_Oligo Pdgfra
057_L5/6 IT TPE−ENT Chn2
076_L6b CTX Nxph4
028_Sst Calb2
063_L5 PT CTX Npnt
027_Sst Hpse
ACA
AId−AIv
AId−AIv−AIp
AUD
ENT
HIP
MOp
MOs−FRP
PAR−POST−PRE−SUB−ProS
PL−ILA−ORB
PTLp
RSP
SSp
SSs−GU−VISC−AIp
TEa−PERI−ECT
VIS
VISl
VISm
VISp
Dissection

Supplementary Figure 3: Disease diagnosis and treatment data. A) Dot plot showing the fraction of male vs. female individuals with each diagnosis (rows sum to 1). Blue arrow indicates the subset of data points used in panel **B**. **B**) River plot showing the relationship between gender, treatment category, and treatment subcategory for individuals diagnosed with "Prevention/Good Health". **C**) Dot plot showing the fraction of individuals of different ages with each diagnosis. Legend from **A** applies but note the ordering of diagnoses is different. Purple arrows indicate the subset of data points used in panels **D** and **E**. **D-E**) Dot plot showing the relationships between disease subcategory and treatment category for individuals diagnosed with "Mental" (**D**) or "Central Nervous System (CNS) / Neuromuscular" (**E**). Fraction of individuals in each treatment category use the same legend in both panels (rows sum to 1). Similarly, dots are color-coded by treatment category to further allow linking between panels.

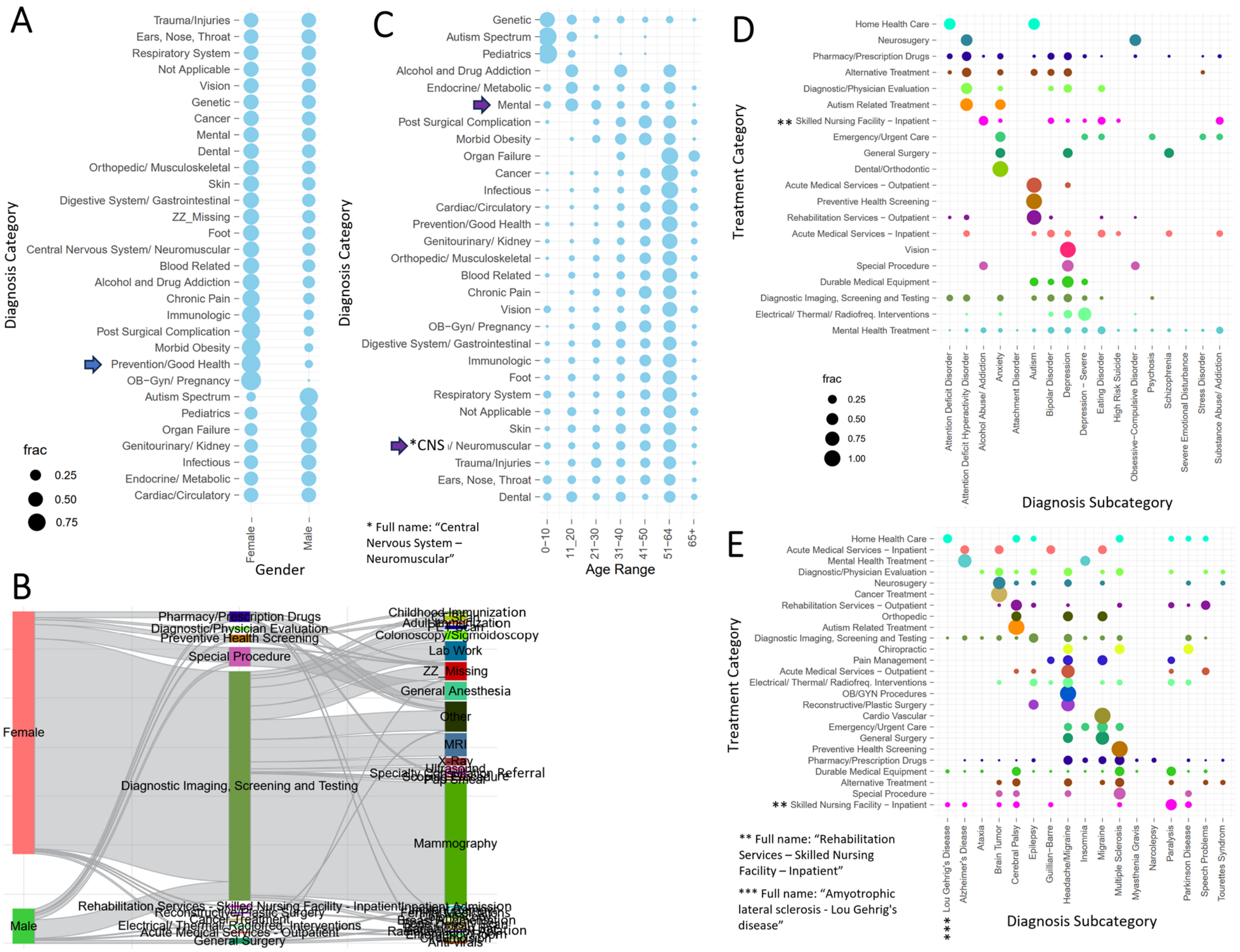

Supplementary Figure 4: Comparison of US county-level population data. A) Bee swarm plots showing median household income for all US counties identified as metropolitan (Metro) or non-metropolitan (Non-Metro) area, separating and color-coding by metropolitan designation (left plots) and separated out by state (right plots). A few counties with high median income are highlighted. **B-D**) Scatterplots showing the relationship between correlated metrics and color-coded as in **A**. PCTPOVALL_2021 is the estimated percent of people of all ages in poverty in 2021.

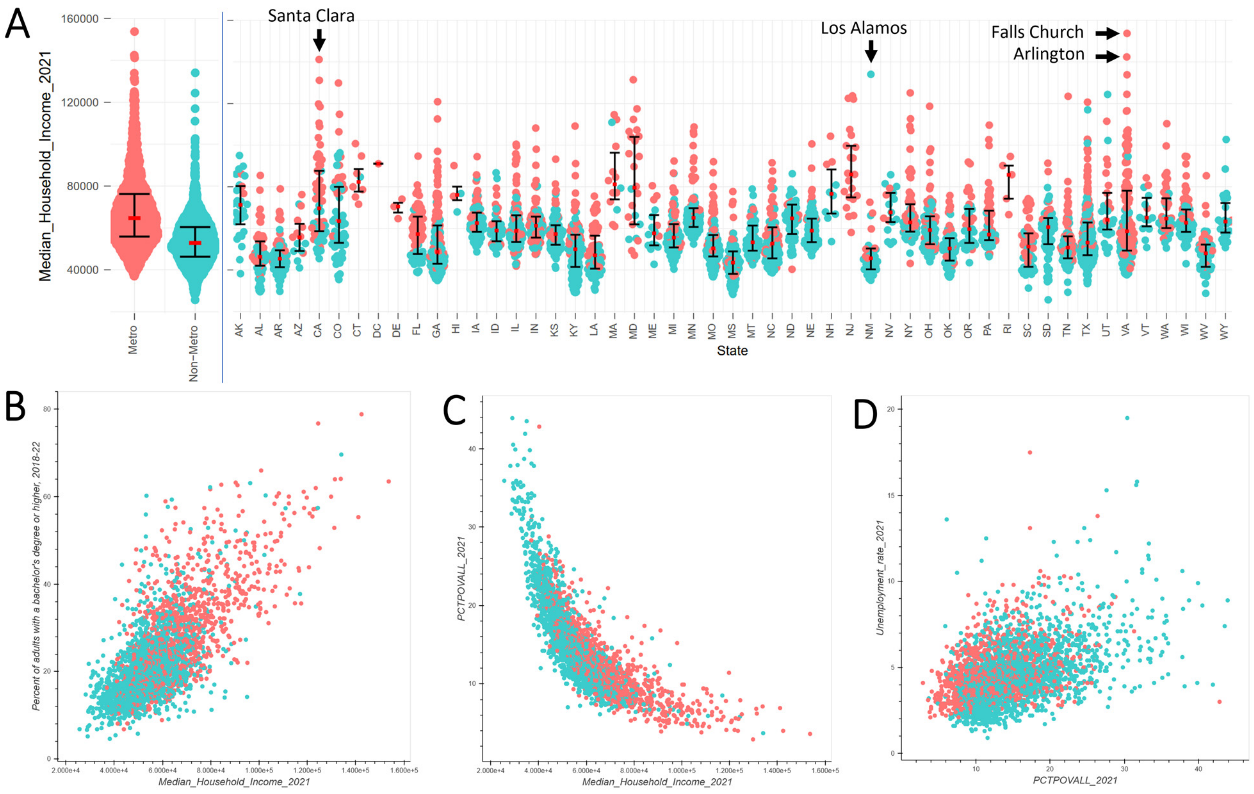

Supplement: Supplement 2 [file media-2.pdf]
